# Supplementary material for: Distal-less and spalt are distal organisers of pierid wing patterns
Source: EvoDevo. 2022 Jun 3;13:12. doi: 10.1186/s13227-022-00197-2 (PMC9164424; doi:10.1186/s13227-022-00197-2)
Supplement: Supplementary file 1 — Additional file 1. List of primers, single-guide RNA sequences and buffers used in this study. [file 13227_2022_197_MOESM1_ESM.docx]

Additional Files

Table S1. **A summary of all the primers used for *in-situ* hybridisations and CRISPR/Cas9 experiments.**

| Primers ID | Sequence |
| --- | --- |
| *salm (P. canidia)* Forward Primer | TTTCAGTAGCAGGGCATGTG |
| *salm (P. canidia)*  Reverse Primer | GATGGTGCACGTTGTGTTTC |
| *salm sgRNA 1* | GAAATTAATACGACTCACTATAGGTGCGGTTGGCGGTTTCGGGAGTTTTAGAGCTAGAAATAGC |
| *dpp (P. canidia)* Forward Primer | ACCACACCGCTACAGACCTC |
| *dpp (P. canidia)* Reverse Primer | GCACCACATTGTTCACTTCG |
| *Dll (P. canidia)* Forward Primer | AAGCGGTGAAAATCACAACC |
| *Dll (P. canidia)* Reverse Primer | TCTGGTAGAGCCAGGTACTGC |
| *Dll sgRNA 1* | GAAATTAATACGACTCACTATAGGCTTAGATGAGACATTCGTGGGTTTTAGAGCTAGAAATAGC |
| *Dll sgRNA 2* | GAAATTAATACGACTCACTATAGGGTAGATGGTGGAAGACTGCGGTTTTAGAGCTAGAAATAGC |

**Table S2. Composition of buffers used in immunostaining reactions.**

| **10X Phosphate-buffered saline (PBS) (In 500mL)** | |
| --- | --- |
| Dipotassium hydrogen phosphate (K_2_HPO_4_) | 5.34 g |
| Potassium dihydrogen phosphate (KH_2_PO_4_) | 2.64 g |
| Sodium chloride (NaCl) | 40.9 g |
| Milli-Q Water | To 500 mL |
| **Fix Buffer (In 30 mL)** | |
| 500 mM PIPES (C_8_H_18_N_2_O_6_S_2_) pH 6.9 | 6 mL |
| 500 mM EGTA (C_14_H_24_N_2_O_10_) pH 6.9 | 60 μL |
| 20% Triton™ X-100 | 1.5 mL |
| 1 M Magnesium sulfate (MgSO_4_) | 60 μL |
| Milli-Q Water | 22.4 mL |
| 37% Formaldehyde (CH_2_O) | Add 55 μL per 500 μL of Fix Buffer |
| **Block Buffer (In 40 mL)** | |
| 1 M Tris-HCl pH 6.8 | 2 mL |
| 5 M Sodium chloride (NaCl) | 1.2 mL |
| 5 mg/mL Bovine Serum Albumin (BSA) | 0.2 g |
| Milli-Q Water | 35.8 mL |
| **Wash Buffer (In 200 mL)** | |
| 1 M Tris-HCl pH 6.8 | 10 mL |
| 5 M Sodium chloride (NaCl) | 6 mL |
| 20% IGEPAL-CA630 | 5 mL |
| 1 mg/mL Bovine Serum Albumin (BSA) | 0.2 g |
| Milli-Q Water | 179 mL |
| **Mounting media** | |
| Tris-HCl pH 8.0 | 20 mM |
| N-propyl gallate | 0.5% |
| Glycerol | 60% |

**Table S3. Composition of buffers used in *in-situ* hybridisation reactions**.

| **10X Phosphate-buffered saline (PBS) (In 500 mL)** | |
| --- | --- |
| Dipotassium hydrogen phosphate (K_2_HPO_4_) | 5.34 g |
| Potassium dihydrogen phosphate (KH_2_PO_4_) | 2.64 g |
| Sodium chloride (NaCl) | 40.9 g |
| RNase-free water | To 500 mL |
| **1X Phosphate-Buffered Saline, 0.1% Tween® 20 Detergent (PBST) (In 50 mL)** | |
| 1X Phosphate-buffered saline (PBS) | 50 mL |
| Tween® 20 | 50 μL |
| **20X Saline Sodium Citrate Buffer (SSC) (In 1000 mL)** | |
| 3 M Sodium Citrate (NaCl) | 175.3 g |
| Trisodium citrate (Na_3_C_6_H_5_O_7_) | 88.2 g |
| RNase-free water | 800 mL |
| Use 1 M Hydrochloric acid (HCl) to adjust pH to 7.0 Make up volume of buffer to 1 L using RNase-free water Autoclave the buffer before use |  |
| **Pre-hybridisation buffer (In 40 mL)** | |
| Formamide (CH_3_NO) | 20 mL |
| 20X Saline Sodium Citrate Buffer (SSC) | 10 mL |
| Tween® 20 | 40 μL |
| RNase-free water | 10 mL |
| **Hybridisation Buffer (In 40 mL)** | |
| Formamide (CH_3_NO) | 20 mL |
| 20X Saline Sodium Citrate Buffer (SSC) | 10 mL |
| Tween® 20 | 40 μL |
| Salmon Sperm DNA | 40 μL |
| Glycine (C_2_H_5_NO_2_) (100 mg/mL) | 40 μL |
| RNase-free water | 10 mL |
| **Block Buffer (In 50 mL)** | |
| 1X Phosphate-buffered saline (PBS) | 50 mL |
| Tween® 20 | 50 μL |
| Bovine Serum Albumin (BSA) | 0.1 g |
| **Alkaline phosphatase buffer (In 20 mL)** | |
| Tris Hydrochloride (Tris-HCl) (pH 8.0) | 2 mL |
| 5 M Sodium chloride (NaCl) | 400 μL |
| 200 mM Magnesium chloride (MgCl_2_) | 250 μL |
| Tween® 20 | 20 μL |
| RNase-free water | To 20 mL |

Table S4. **Summary of CRISPR/Cas9 experiments for *Distal-less* and *spalt* knockouts.**

| ***Distal-less*** | | | |  |  |
| --- | --- | --- | --- | --- | --- |
| Concentration of sgRNA: Cas9 protein | Eggs Injected | Hatchlings | Hatch Rate | No of adults with mutant phenotypes | Description of phenotypes observed |
| 100 ng/μL: 300 ng/μL in 10 μL | 357 | 70 | 19.6 | 3 | Missing melanic scales along wing margin in both forewings and hindwings |
| ***spalt*** | | | |  |  |
| Concentration of sgRNA: Cas9 protein | Eggs Injected | Hatchlings | Hatch Rate | No of adults with mutant phenotypes | Description of phenotypes observed |
| 300 ng/μL: 600 ng/μL in 10 μL | 575 | 98 | 17.0 | 8 | Disruption of Cu2 vein, missing melanic scales in spot region and along the wing margin in forewings, lightened spot markings |

**Probe sequence of *B. anynana* *dpp* used for in situ hybridisation.**

GTTCTTCAACGTAAGCGGCGTACCGGCCGACGAGGTGGCGCGCGGCGCCGACCTCTCGTTCCAACGAGCCGTCGGCACCACCGGCAGACAGAGACTGTTGTTGTACGACGTGGTGCGCCCTGGCCGCCGCGGCCACTCCGAGCCGATCCTGCGGCTGCTGGACTCCGTTCCGCTCCGGCCCGGGGAGGGAATCGTCAACGCCGACGCTCTGGGAGCGGCGCGACGGTGGCTCAAAGAGCCCAAACATAATCACGGACTATTAGTGCGAGTGTTAGAAGAAGACGCCGCGAGTGCGAGCAGGGACGCGAAGTTCCCGCACGTGCGCGTGCGCAGACGCGTCACGGACGAGGAGGAGGAGTGGCGGACGGCGCAGCCGCTGCTCATGCTGTACACGGAGGACGAGCGCGCGCGCGCGTCGCGGGAGACGAGCGAGCGGCTGACGCGCAGCAAGCGCGCGGCGCAGCGGCGGGGGCACCGCGCGCACCACCGCCGCAAGGAGGCGCGCGAGATCTGCCAGCGCCGCCCGCTGTTCGTCGACTTCGCGGACGTGGGCTGGAGCGACTGGATCGTGGCCCCGCACGGCTACGACGCGTACTACTGCCAGGGCGACTGCCCCTTCCCGCTGCCGGACCACCTCAACGGCACGAACCACGCGATAGTGCAGACTCTGGTCAACTCAGTGAACCCCGCGACGGTGCCCAAAGCGTGCTGCGTGCCGACGCAACTCTCATCTATATCTATGTTATATATGGACGAAGTGAACAATGTGGTGCTTAAAAACTATCAGGACATGATGGTGGTAGGCTGTGG

**Probe sequence of *P. canidia dpp* used for in situ hybridisation.**

ACCACACCGCTACAGACCTCGACGATCGCTTCCCTCAGGAGCATCGCTTTCGCCTATATTTCAACATAAGTGGCGTACCTGGCGACGAAGTCGCTCGAGGCGCGGATGTCACCTTTCAACGCGCCGTCGGTGTCACCGGCACACAGAGGCTGCTGCTGTACGACGTGGTGCGCCCGGGCAGACGAGGAAAGAGCGAACCCATTTTGAGACTCCTCGATTCCATTCCGCTCCGACCCGGCCAAGGTTCGGTCGCGGCCGACGCCCTCAGCGCGGCGAGAAGGTGGCTCAAGGAACCGCAACATAATCATGGCCTATTAGTGCGCGTCATAGACGATACCGTAGGCAATGAAAGTGTAAAATTTCCACATATTCGCGTCCGACGGCGCGCTACAGACGAGCACGAGGAATGGAGCGCCATCCAGCCTCTGCTGATGCTTTACACGGAGGATGCGAGAGCGAGAACGGCTCGGGAGCGTGGAGAGTCGTCGCTGACGAGAAATAAGAGAGCGACGCAGCGGAAGGGCCACCGGCCTCACCACAGGCGTAAGGAGGCGCGGGAGATCTGCCAGAGGCGCCCCCTGTTCGTGGATTTCGCGGACGTGGGTTGGAGTGACTGGATTGTCGCCCCCCAGGGCTACGAAGCCTACTATTGCCAGGGCGATTGCCCCTTCCCATTAGCCGATCACCTCAATGGTACGAACCATGCGATTGTGCAGACTTTAGTGAACTCAGTGAATCCGGCCGCGGTGCCGAAGGCGTGTTGTGTGCCGACGCAACTTTCCCCTATATCTATGTTGTATATGGACGAAGTGAACAATGTGGTGC

**Sequence of *P. canidia Distal-less* and site of CRISPR targets.**

ATGGAGCGAGAGGCTCACAAAGCGGTGAAAATCACAACCAAGCATCCGAAATCCCTCAAAATTACCCGAATTCAATCCCCAAACACAAAACCGGCCACGCTGAGTTTCTCAGATCCCTTCGGGCCTCCCCAGTCCGCGGACGGGGGGGGCCCATCAACCCCCCAACCAGCCATGACCACCCAAGAGGCGTTGGAGCACCAGCACCACCATTTGGGGGGCACGCAAACCCCCCACGACATCTCGAACTCCGCCAATTCCACCCCCACGAATGTCTCATCTAAGTCCGCGTTCATCGAGCTTCAACAGCATGGGTATGGGTTCAAGGGGGGCTACCAGCATCCCCACCATTTTGGGAGTCCGGGGGGACAACAGAACCCTCATGAAGCGTCGGGATTCCCCAGTCCTAGATCGTTAGGTTACCCCTTCCCTCCCATGCACCAGAATACCTATGGTTATCATTTAGGTTCCTATGCCCCCCAATGCGCGAGTCCTCCTAAAGATGAAAAATGTGGCCTCTCCGATGACCCCGGCTTACGGGTAAATGGAAAGGGCAAGAAGATGAGGAAACCCCGCAGTCTTCCACCATCTACTCAGCTTCAGCAGCTTAATAGGCGGTTTCAAAGAACGCAGTACCTGGCTCTACCAGAGAGAGCAGAGCTCGCGGCTAGCTTAGGATTAACGCAGACACAGGTAAGTGGCTTAACAATAAAGAAA

**Sequence of *P. canidia spalt-major* and site of CRISPR targets.**

TTTCAGTAGCAGGGCATGTGACACTAGAAGCACTTCAAAATACGAAGGTTGCCGTAGCACAATTTGCTGCAACGGCAATGGCCAATAACGCCAACAATGAAGCTGCTTTACATGAGCTGGCAGTCTTACAGAGTACGTTGTTCACATTGCAGCATCAGCAAGTGTTTCAACTTCAATTAATAAGACAGTTGCAAAATCAATTATCATTAACGAGAAGAAAAGATGATCAACCACCAAGTCCATCGCCGGTTGAACAAGAAGCGACCGCTCCATCGACTCCGGTTCGATCACCATCACCGCCTCGTCCGCCACGGGAGCCATCTCCTGCTGCACCAACTCCTCCCAGTAGCCAAAGCTTGCCATCGACCCACTCGCATATCACACCTAAAATTGAACCGATTTCCATCCCGAAACCGCCAACCGCATCTCCACCTATGATGTCACATCCACCCTACAGCTCCATTTCGTCTTCATTAGCTTCTTGTATTATCACGAATAATGATCCTCCACCGTCCCTTAATGAACCAAATACACTTGAAATGCTACAGAAGCGAGCGCAAGAAGTACTTGACAATGCATCACAAGGTTTATTAGCAAATAATTTAGCCGACGAACTGGCGTTTAGAAAATCTGGTAAAATGTCACCCTATGATGGAAAAAGTGGAGGTAGAAATGAACCGTTTTTCAAGCACAGATGCAGATATTGTGGAAAAGTTTTTGGAAGTGACTCTGCACTCCAAATACACATACGGTCACATACAGGCGAGCGACCTTTTAAATGTAATGTTTGTGGATCAAGATTCACAACAAAAGGAAACCTTAAAGTCCACTTTCAAAGGCATACATCTAAGTTTCCACACGTAAAAATGAACCCGAACCCAGTGCCAGAACACTTAGACAAATACCACCCCCCACTACTTGCACAACTATCTCCGGGGCCAATTCCAGGGATGCCCCCACATCCTCTTCAGTTTCCTCCTGGCGCACCAGCTCCATTTCCGCCAAGCTTGCCATTATACAGACCAACGCATCATGATTTACTTCCCCCTCGCCCACTCGGTGACAAGACACTTCCACCACACCCATTATTTACAATGAGAGAAGAGCAAGATGCACCTGCAGATTTAAGCAAACCTTCTGCACCCAGCCCATCAAGATTAACATCTGAGATGTTTAAGTCTGAGCCACAAGACGATGAGAGCCAACGCGATTCTAGTTTTGAAGAAACTGACCGAATATCACCTAAGCGAGAGCCAGAGGAGAATGAACCCGTACATGACGCAGAACAAGATCGATATCCATCCACTTCACCCTACGATGACTGCAGTATGGACTCGAAGTATAGTAATGAAGACCAAATCGGAAGAGAGAGCCCTCACGTGAAGCCGGATCCTGATCAACCGGAAAATCTTTCAAGTAAGAATCGACCGGGCAGCAACGATAACTCATGGGAAAGTTTAATTGAAATAACGAAAACTTCAGAAACATCCAAGCTACAGCAATTAGTTGACAATATTGACAATAAGGTGTCTGATCCAAATGAATGTATTGTGTGTCATCGTGTTCTTTCTTGTAAAAGTGCTTTGCAAATGCACTACCGTACTCACACCGGTGAGAGACCATTTCGTTGTAAATTATGTGGTCGAGCATTTACTACTAAAGGAAATCTTAAAACCCATATGGGTGTTCACCGCATTAAACCTCCTTCTCAAATTTTACACCAATGTCCTGTTTGCCATAGAAGGTTTCCTGATCCGAATATTCTCCATCAACACATTCGAACACACACAAGCGACCGTTACAGTACCCCTTTCGATCAATTAATGATTCGCGACTTAACCGACAGTCAATCAATAAGCAATAATGACTCTGAATATGTGCGTGGAAACACAACGTGCACCATC
